# Supplementary material for: A variant NuRD complex containing PWWP2A/B excludes MBD2/3 to regulate transcription at active genes
Source: Nat Commun. 2018 Sep 18;9:3798. doi: 10.1038/s41467-018-06235-9 (PMC6143588; doi:10.1038/s41467-018-06235-9)
Supplement: Supplementary file 1 — Supplementary Information [file 41467_2018_6235_MOESM1_ESM.pdf]

A variant NuRD complex containing PWWP2A/B excludes MBD2/3 to regulate transcription at active genes

Zhang *et al.*

Contents

Supplementary Figures 1-7

Supplementary Tables 1-2

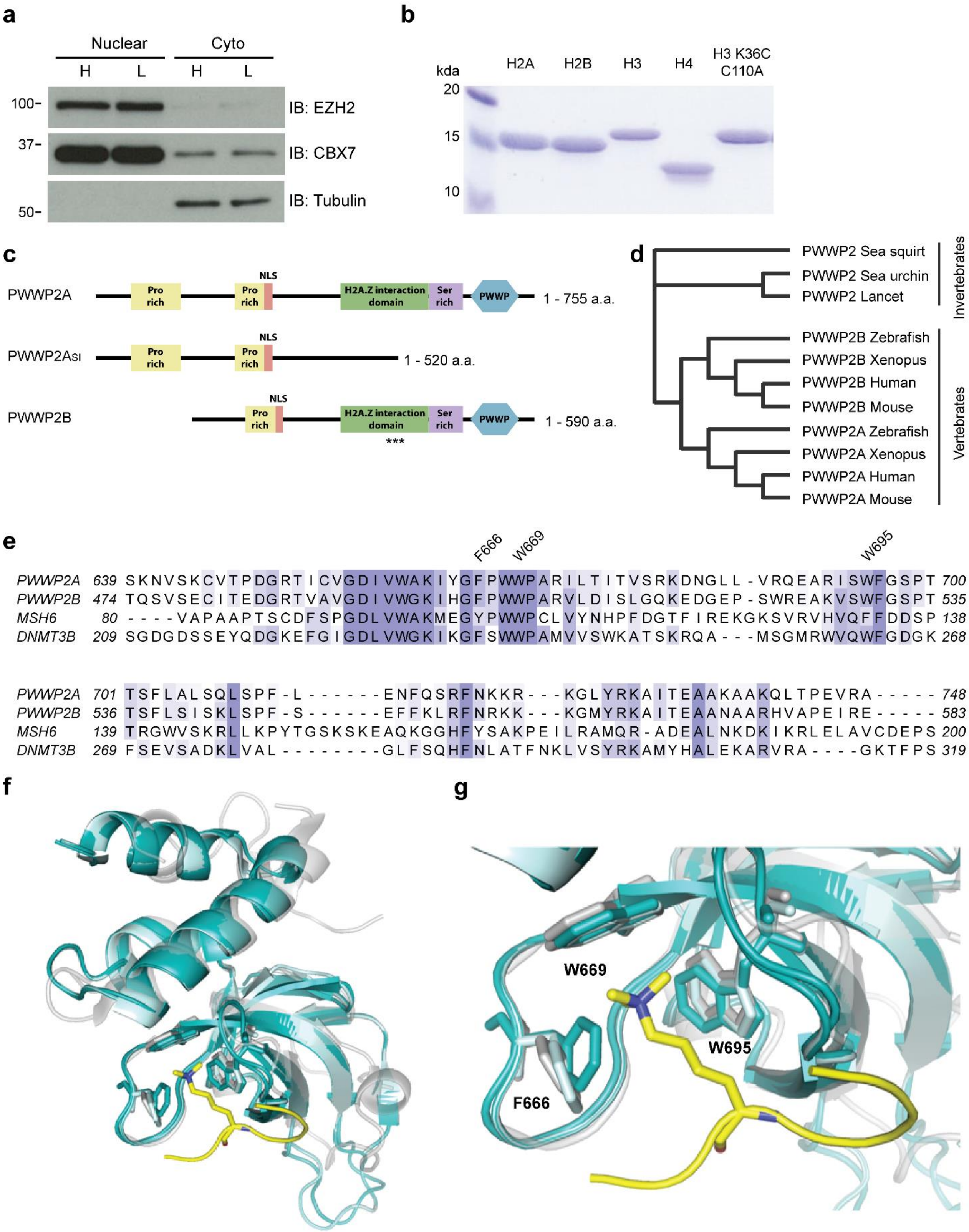

Supplementary Figure 1. PWWP2A/B proteins recognise H3K36me3

(a) Immunoblot of nuclear (EZH2, CBX7) and cytoplasmic (Tubulin) proteins in the nuclear and cytoplasmic fractions of heavy (H) and light (L) SILAC-labelled cells.

(b) Recombinant purified histones H2A, H2B, H3, H4, and H3 K36C C110A.

(c) Domain structure of the PWWP2A long isoform, PWWP2A short isoform, and paralog PWWP2B. *** H2A.Z interaction domain in PWWP2B based on sequence homology.

(d) PWWP2A is evolutionarily conserved in chordates. Paralogs PWWP2A and PWWP2B likely result from a gene duplication event in early vertebrate evolution.

(e) Alignment of the PWWP domain of PWWP2A and PWWP2B with the PWWP domains of two H3K36me3 readers MSH6 and DNMT3B. Residues which form the H3K36me3-binding aromatic cage in MSH6 and DNMT3B are conserved in PWWP2A/B (F666, W669, W695).

(f) Alignment of the crystal structure the PWWP domain of DNMT3B (grey) bound to an H3K36me3 peptide (yellow) (PDB 5CIU) with the PWWP domain of PWWP2B (PDB 4LD6, teal), and a model of the PWWP domain of PWWP2A (light blue) based on the crystal structure from PWWP2B.

(g) Close up of the aromatic cage and residues involved in H3K36me3 binding (F666, W669, W695 in PWWP2A). DNMT3B in grey, PWWP2A in light blue, PWWP2B in teal, and the H3K36me3 peptide in yellow.

a

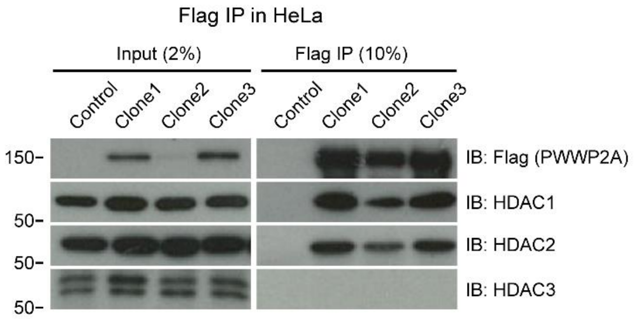

b

PWWP2A interactors in HeLa

	Protein	ID score (Mascot)	Significant matches	Significant sequences
1	PWWP2A	14324	650	63
2	MTA1.3	5346	208	63
3	MTA1	5004	194	59
4	MTA2	2307	85	41
5	MTA3	1765	72	30
6	RBBP7	2074	85	21
7	RBBP4	1489	67	19
8	HDAC2	1650	86	25
9	HDAC1	1562	84	26
10	BEND3	986	26	26
11	PWWP2B	455	20	18

c

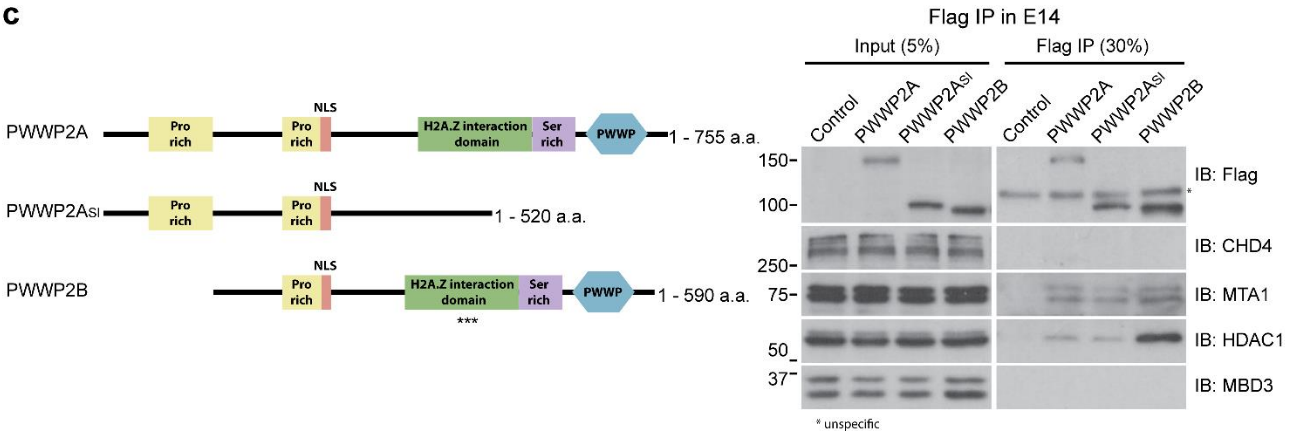

d

PWWP2B interactors in mESCs

	Protein	-10lgP	Significant matches	Significant sequences
1	MTA1	329	27	19
2	MTA3	294	19	11
3	MTA2	237	14	11
4	RBBP4	189	9	4
5	RBBP7	180	9	4
6	HDAC1	170	7	3
7	HDAC2	155	6	2
8	PWWP2A	95	2	2

Supplementary Figure 2. PWWP2A/B interacts with the HDAC subcomplex of NuRD

(a) FS2-PWWP2A co-immunoprecipitates HDAC1 and HDAC2 but not HDAC3 in HeLa cells.

(b) Identification of PWWP2A associated proteins in HeLa cells by LC-MS/MS and identification by Mascot (0.01 FDR). (also see Supplementary Table 2 and 3)

(c) The PWWP2A long protein isoform, the PWWP2A short protein isoform, and the paralog PWWP2B all co-immunoprecipitate MTA1 and HDAC1, but not CHD4 and MBD3 in mESCs. *** H2A.Z interaction domain in PWWP2B based on sequence homology.

(d) Identification of PWWP2B associated proteins in mESCs by LC-MS/MS and identification by Mascot (0.01 FDR). (also see Supplementary Table 4)

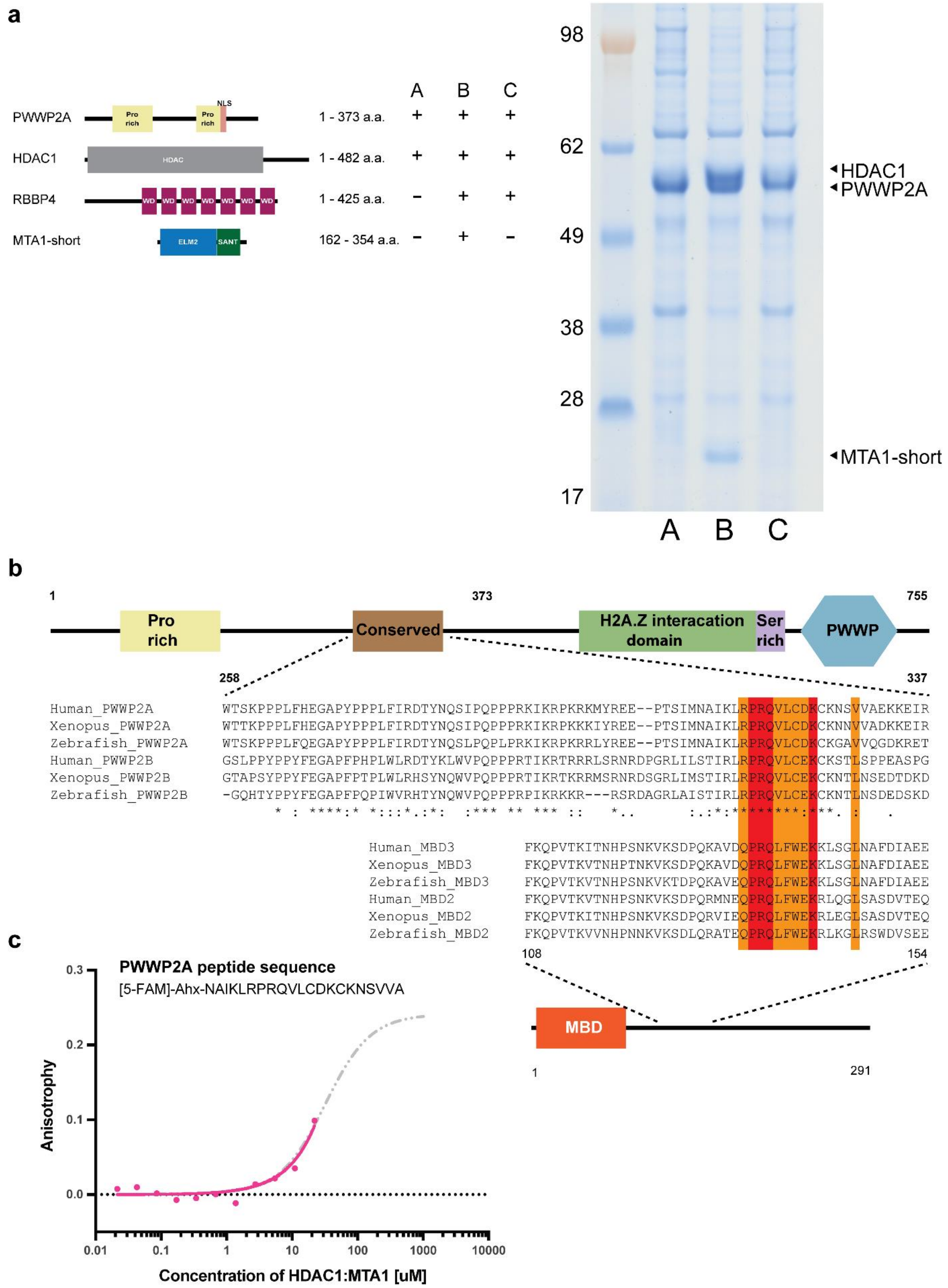

Supplementary Figure 3. PWWP2A forms a stable complex with HDAC1 and and MTA1

(a) Co-expression of Flag-PWWP2A$_{1\text{-}373}$ with HDAC1, RBBP4 and MTA1$_{162\text{-}354}$. A) PWWP2A and HDAC1 fails to interact in the absence of MTA1 B) PWWP2A, HDAC1, and MTA1$_{162\text{-}354}$ form a stoichiometric complex. MTA1$_{162\text{-}354}$ lacks the RBBP4 binding domain (see Fig. 3a,b)

thus RBBP4 is not present in this complex. C) Co-expression of PWWP2A, HDAC1, and RBBP4 in the absence of MTA1 does not result in a complex.

(b) Sequence alignment of PWWP2A and PWWP2B from various species highlighting the conserved region within $PWWP2A_{1\text{-}373}$. A conserved motif (R/QPRQV/I) is also found in MBD2 and MBD3. Identical residues are highlighted in red and conserved residues are highlighted in orange.

(c) Fluorescence anisotropy showed binding of a fluorescein-conjugated PWWP2A peptide to HDAC1:MTA1 complex with binding affinity estimated to be in the range between 20-100 µM. The projected fit for binding at higher concentrations is shown as a grey dotted line.

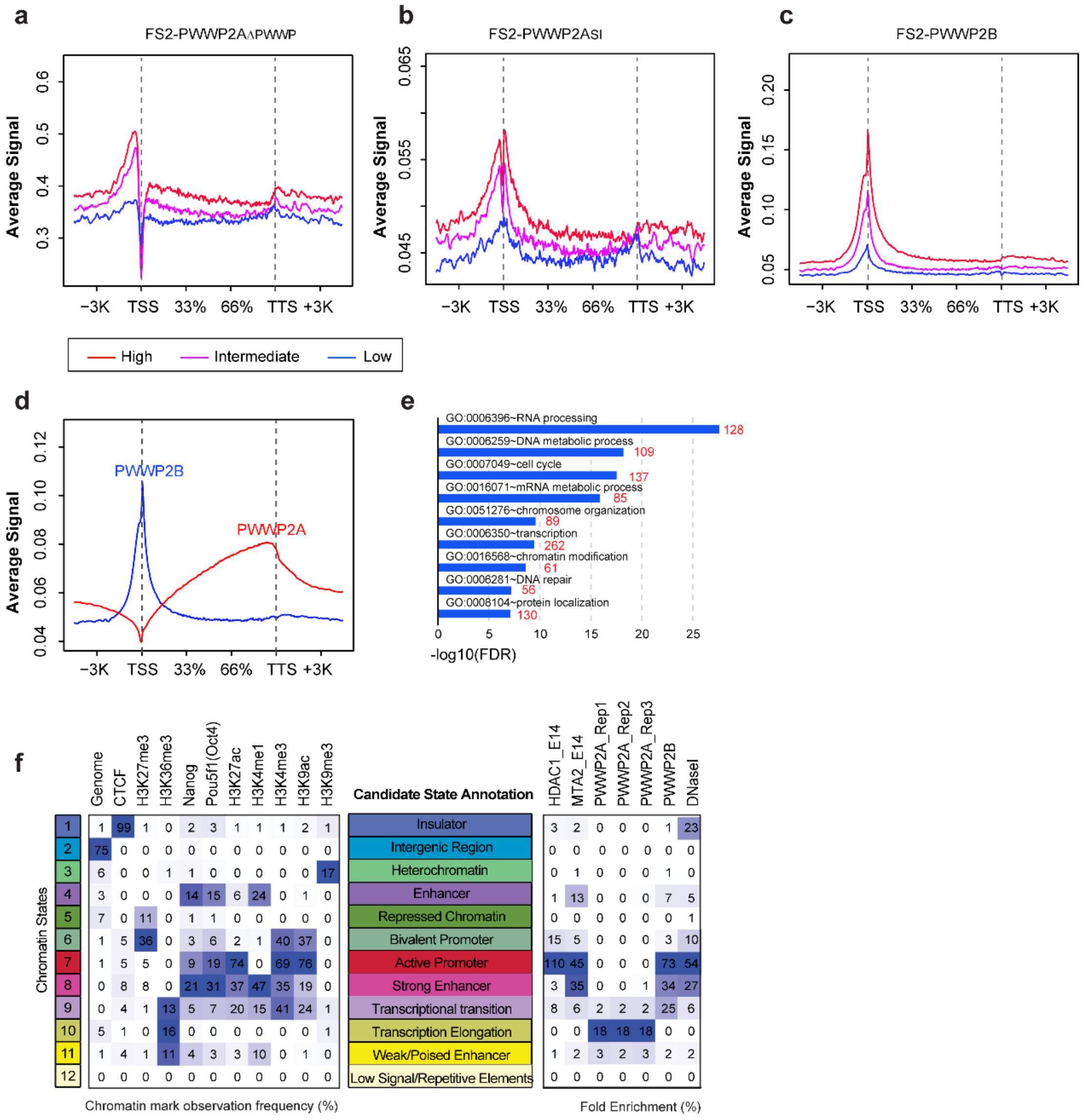

Supplementary Figure 4. Genomic binding pattern of PWWP2A/B in mESCs

(a-c) The binding patterns for PWWP2A$_{\Delta PWWP}$, PWWP2A$_{SI}$ and PWWP2B across high (red), intermediate (purple) and low (blue) expressed genes from 5kb upstream of the TSS to 5kb downstream of the TTS. The x- and y-axis denote the normalized genomic regions and average coverage signals for each categorized gene group.

(d) Meta-gene profile for PWWP2A (red) and PWWP2B (blue) across all gene loci. The x- and y-axis denote the normalized genomic regions and average coverage signals.

(e) Gene Ontology enrichment analysis for PWWP2A high occupancy genes. The length of the bar indicates the significance (-log10(FDR)), the number of genes enriched in each GO term are shown in red.

(f) Chromatin states in mESCs by ChromHMM. The table shows emission parameters learned de novo on the basis of genome-wide recurrent combinations of chromatin marks (left). Each entry denotes the enrichment with which a given mark is found at genomic positions corresponding to the chromatin state.

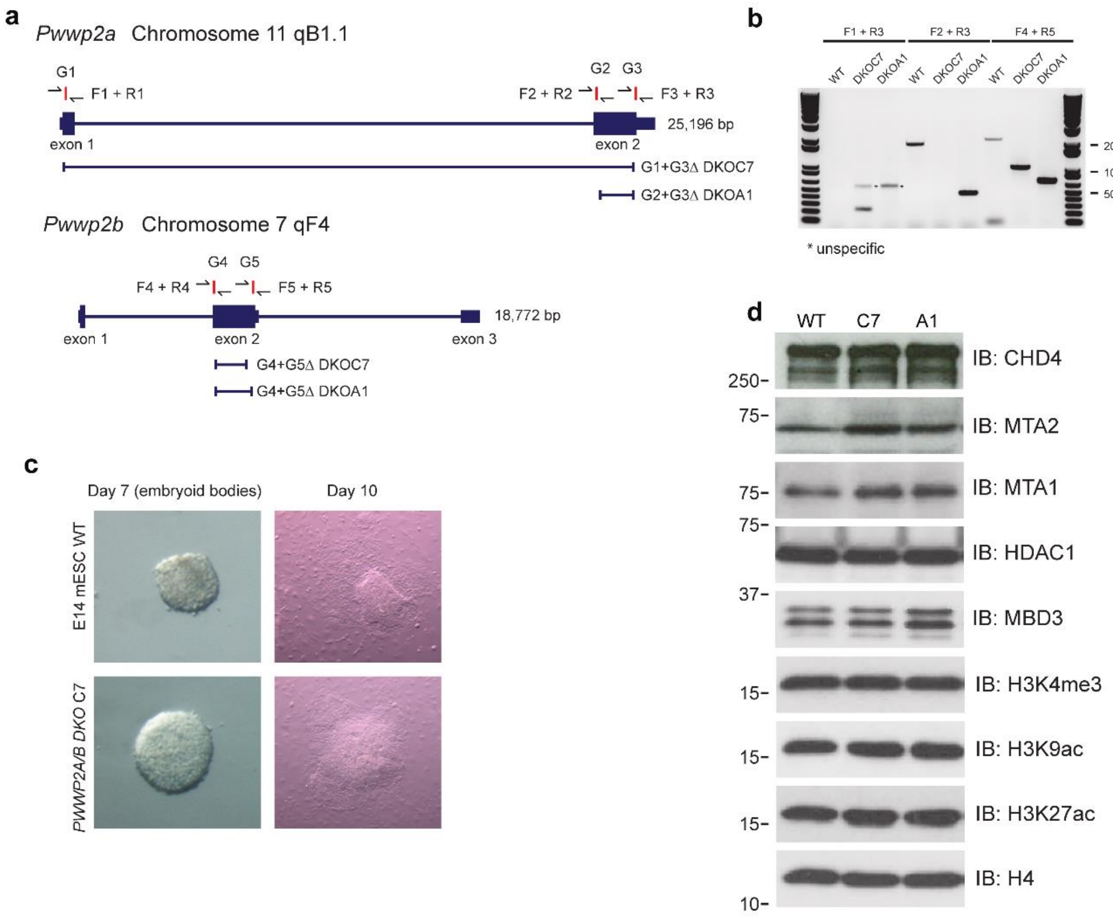
a
Pwwp2a Chromosome 11 qB1.1
G1
F1 + R1
G2 G3
F2 + R2
F3 + R3
25,196 bp
exon 1
exon 2
G1+G3Δ DKOC7
G2+G3Δ DKOA1
Pwwp2b Chromosome 7 qF4
G4 G5
F4 + R4
F5 + R5
18,772 bp
exon 1
exon 2
exon 3
G4+G5Δ DKOC7
G4+G5Δ DKOA1
b
F1 + R3
F2 + R3
F4 + R5
WT
DKOC7
DKOA1
2000
1000
500
* unspecific
c
Day 7 (embryoid bodies)
Day 10
E14 mESC WT
PWWP2A/B DKO C7
d
WT
C7
A1
IB: CHD4
250-
75-
IB: MTA2
75-
IB: MTA1
75-
IB: HDAC1
37-
IB: MBD3
15-
IB: H3K4me3
15-
IB: H3K9ac
15-
IB: H3K27ac
IB: H4
10-

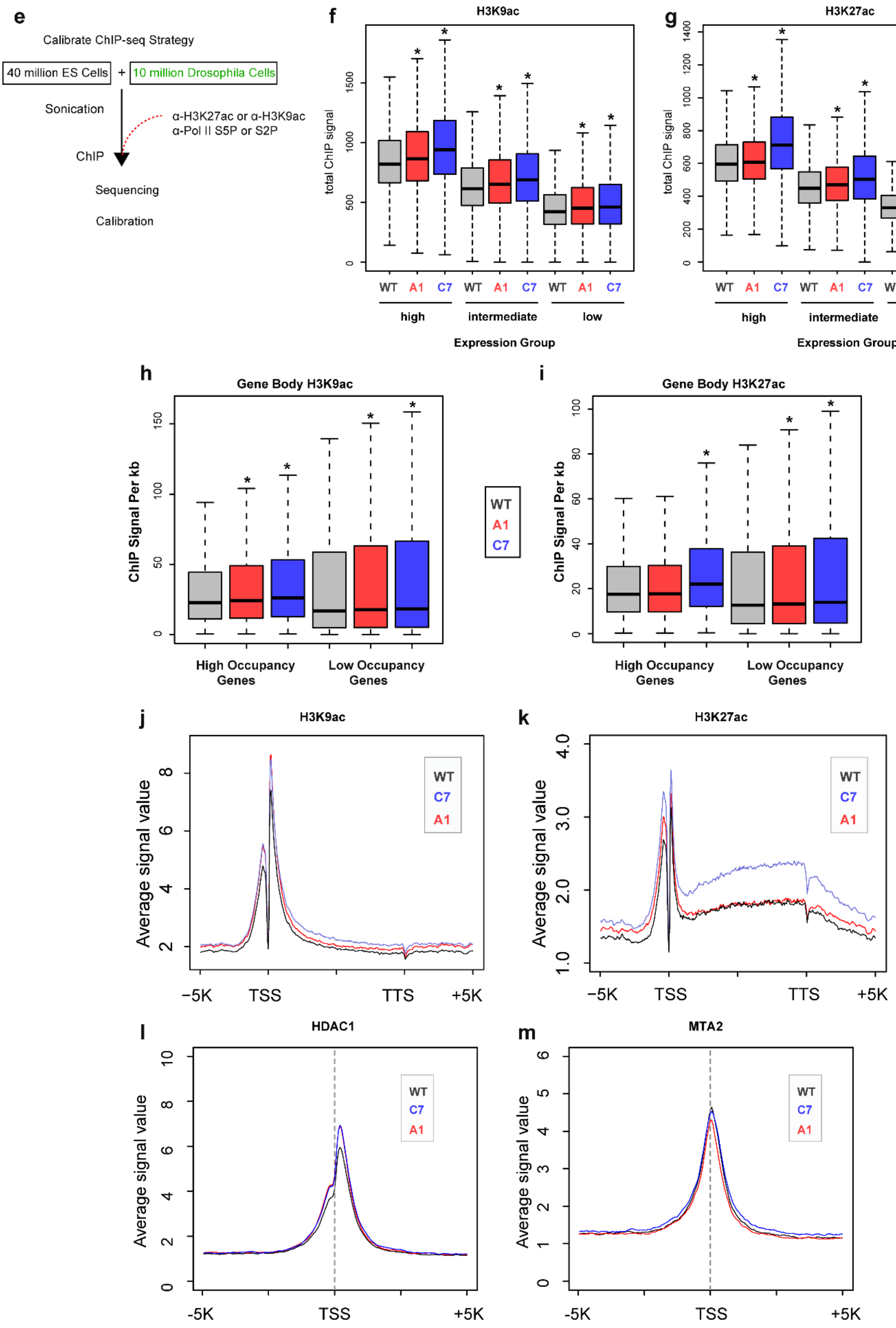

Supplementary Figure 5. Loss of PWWP2A/B leads to an increase in H3K9Ac and H3K27ac

(a-b) Strategy for PWWP2A/B deletion using CRISPR guides (G1-G5). The PWWP2A/B DKO C7 clone was generated with guides G1+G3 which deleted most of exon 1 and exon 2 and

introduced a missense mutation into the PWWP2A gene, and G4+G5 which deleted most of exon2 and introduces a missense mutation into the PWWP2B gene. The DKO A1 clone was generated with guides G2+G3 and G4+G5. Clones were screened for homozygous deletions using different combinations of PCR primers, and deletions were characterised through sequencing.

(c) Differentiation of wildtype and the PWWP2A/B DKO cells under LIF withdrawal conditions. Day 7 – embryoid body formation in suspension. Day 10 – outgrowth of differentiated fibroblast like cells after embryoid bodies are plated onto tissue-culture plates.

(d) Western blot for NuRD components and histone modifications in wildtype and PWWP2A/B DKO cells.

(e) Schematic of the calibrated ChIP-seq procedure. For each sample, 40 million mESCs were combined with 10 million Drosophila cells prior to crosslinking and chromatin preparation. Also see Methods.

(f-g) Box plot showing the ChIP signal for H3K9ac (f) and H3K27ac (g) for WT and DKO cells for genes with high, intermediate, and low expression. Asterisk indicate the significance determined by Mann-whitney U test.

(h-i) Box plot showing the ChIP signal for H3K9ac (h) and H3K27ac (i) for WT and DKO cells for the gene body signal normalised for length (per kb) at high occupancy and low occupancy genes. Asterisk indicate the significance determined by Mann-whitney U test.

(j-k) Calibrated ChIP-seq meta-gene profile for H3K9ac and H3K27ac (average of two biological replicates) at PWWP2A high occupancy genes in WT mESCs and two double knockout cells.

(l-m) Meta gene profiles of the average of two biological replicates for HDAC1 (l) and MTA2 (m) over the region ± 5kb of the TSS for PWWP2A high occupancy genes in WT and PWWP2A/B DKO cells (A1 and C7).

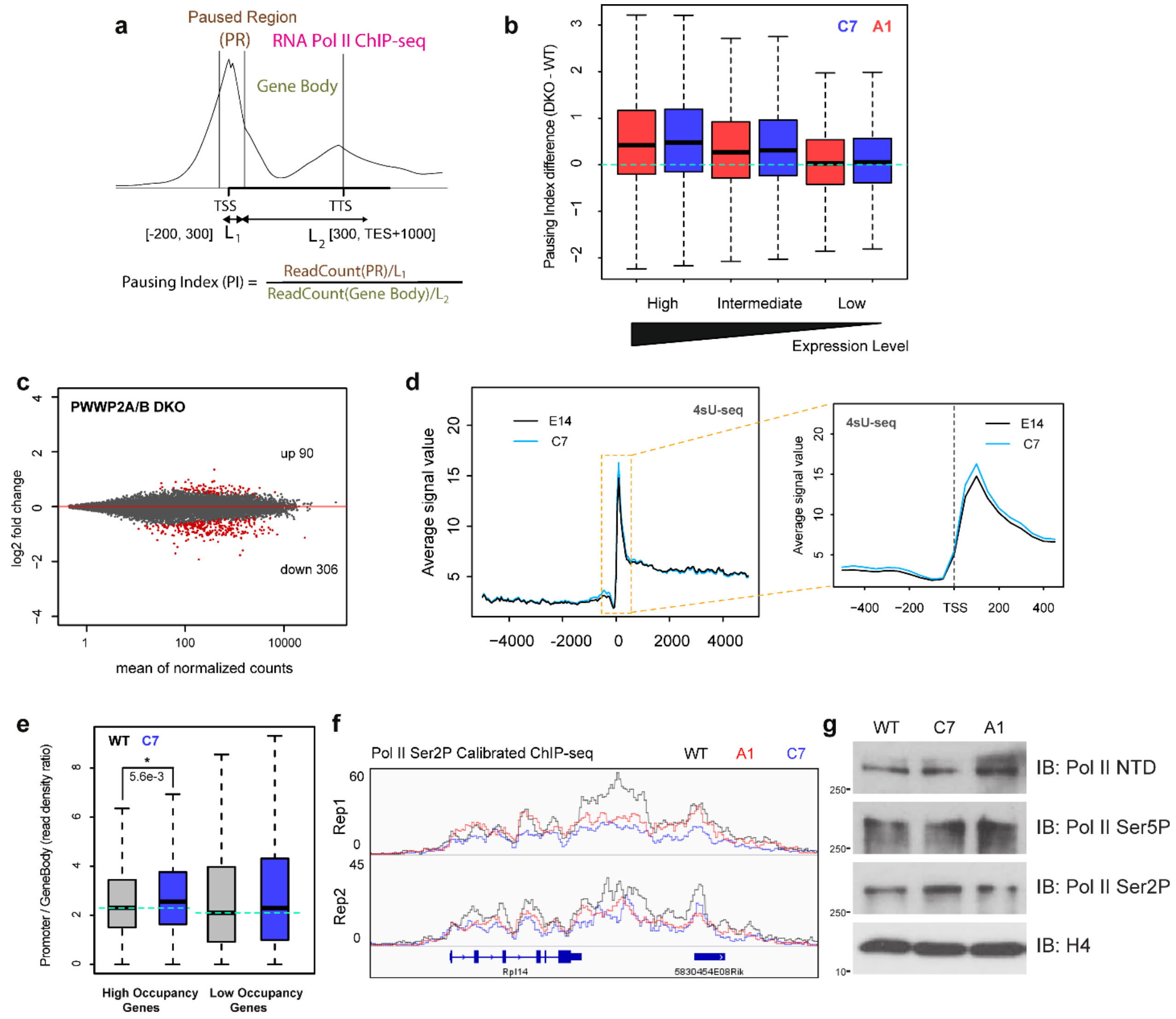

Supplementary Figure 6. Loss of PWWP2A/B leads to Pol II elongation defect at PWWP2A occupancy genes

(a) Calculation of the Pausing Index based on the RNA Pol II NTD ChIP-seq data. The Pausing Index for each gene is the ratio between the RNA Pol II normalised read count at the TSS region over the gene body region.

(b) Boxplot showing the difference in Pausing Index between PWWP2A/B DKO and wildtype for high, intermediate, and low expressed genes.

(c) Differentially expressed genes based on 4sU RNA-seq in WT and PWWP2A/B DKO cell. Red indicates significantly changed genes (adj_p <0.05)

(d) Meta-gene profile for 4sU-seq across the PWWP2A high occupancy genes in wildtype E14 mESCs (black) and PWWP2A/B DKO C7 (blue).

(e) Box plot of the ratio of TSS-proximal to Gene Body read density for PWWP2A high occupancy and low occupancy genes. Significance is labelled and indicated by asterisk.

(f) IGV snapshot showing the *Rpl14* locus for the RNA Pol Ser2P calibrated ChIP-seq. WT is shown in black and the double knockouts in C7 and A1 are shown in blue and red respectively.

(g) Western blot for RNA Pol II NTD (total), Pol II Ser5P and Ser2P in wildtype mESCs and PWWP2A/B DKO cells.

Uncropped westerns

Fig. 2b

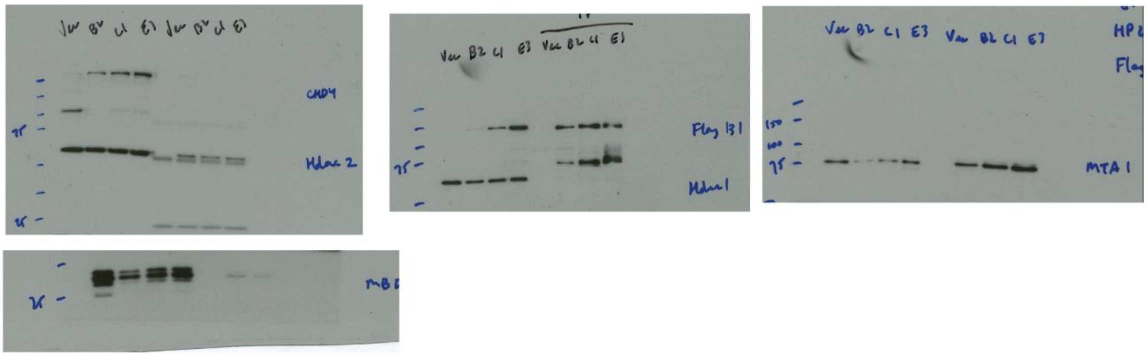

Fig. 2c

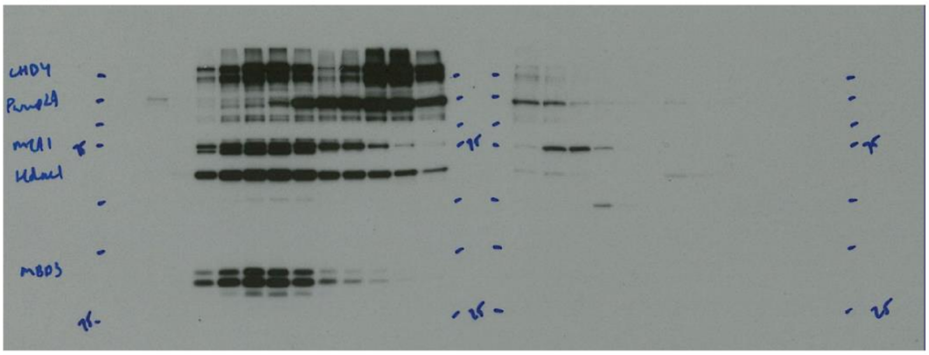

Fig. 2d

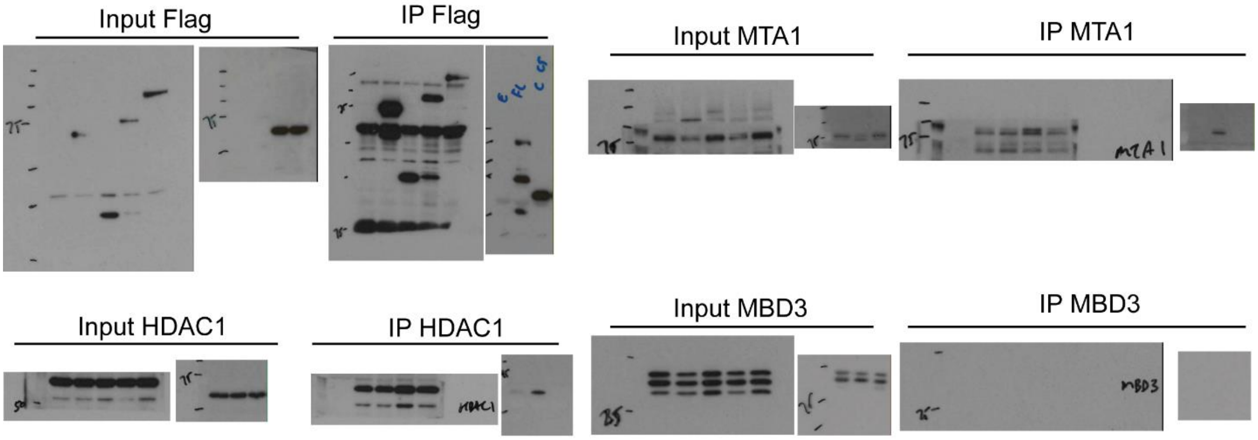

Order: empty vector, 1-373, 148-373, 148-649, ΔPWWP, empty vector, full length, 383-755

Supplementary Fig. 2b

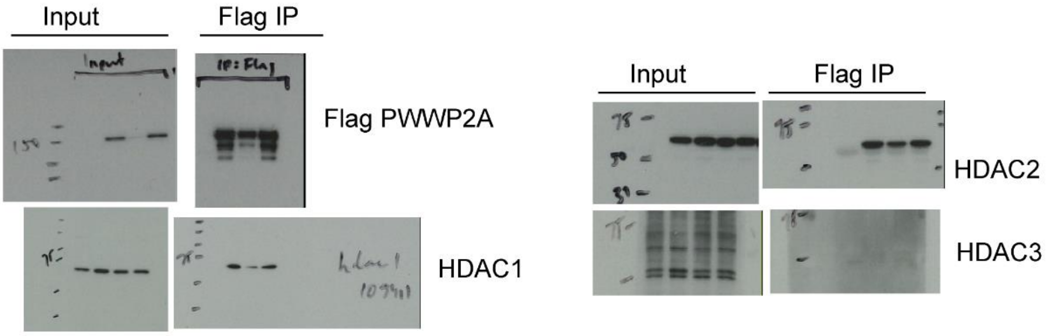

Order: empty vector, clone1, clone2, clone3

Supplementary Fig. 2c

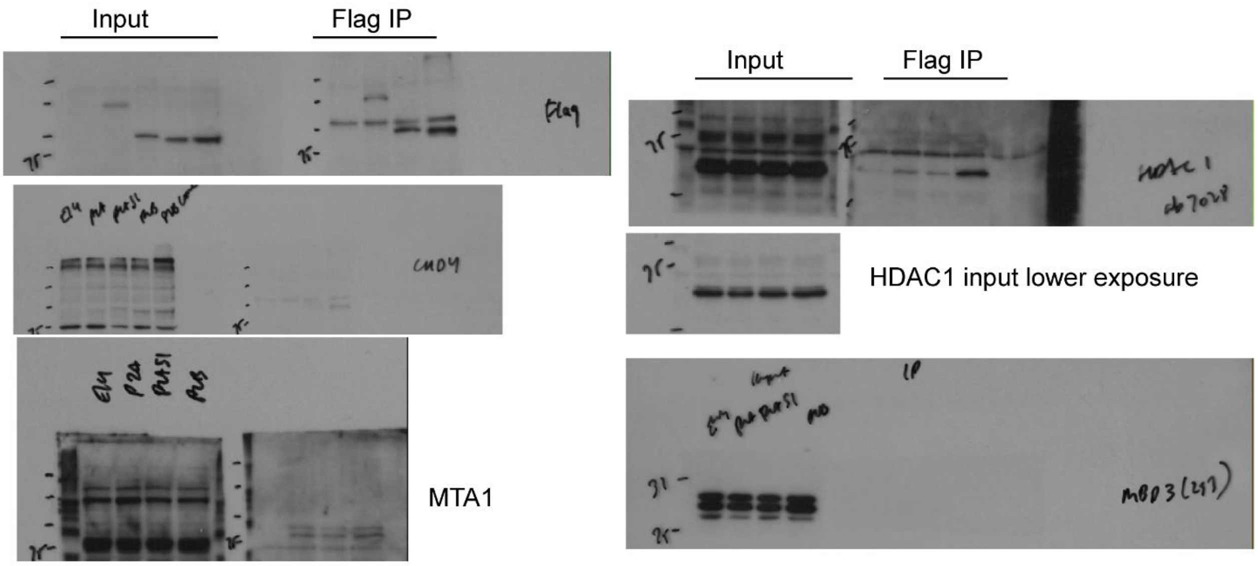

Supplementary Fig. 5d

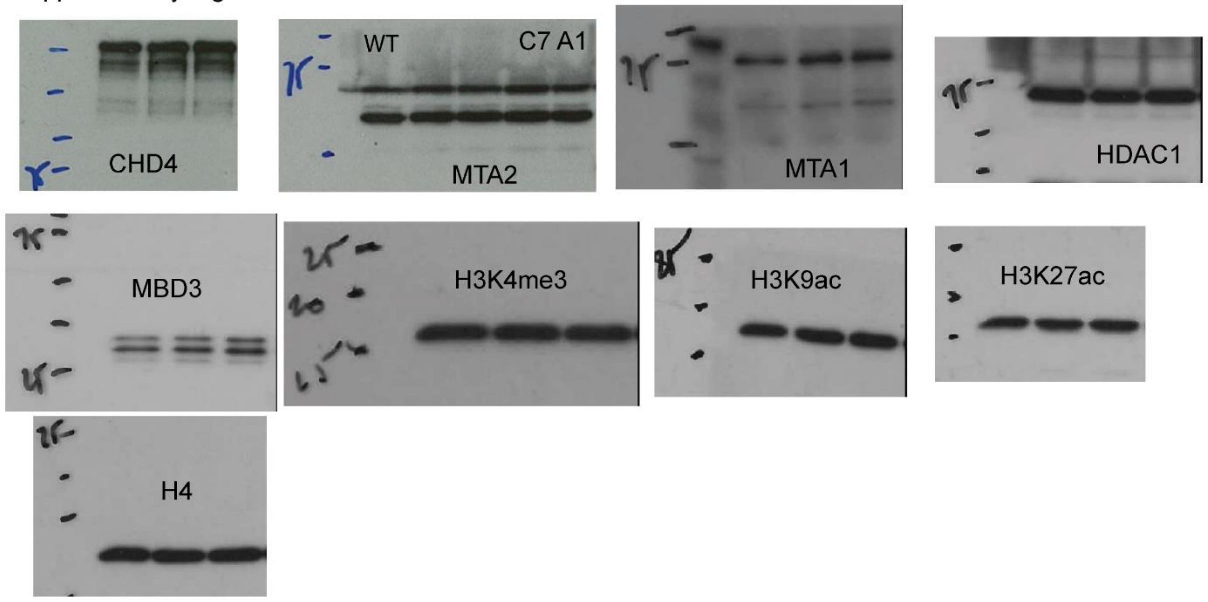

Supplementary Fig. 6

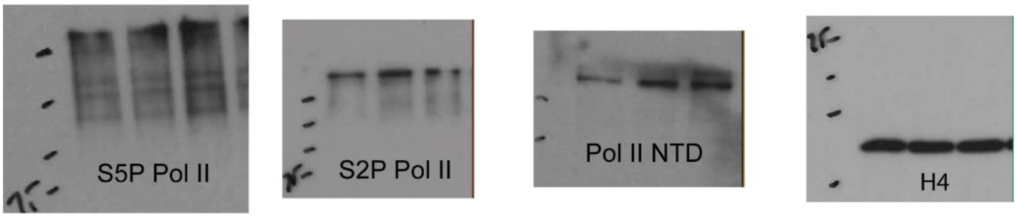

Supplementary Figure 7. Uncropped westerns

Supplementary Table 1. Antibodies

Antibody	Catalogue No.	Company	Usage
EZH2	#5246	Cell Signalling	WB 1:1000
CBX7	Ab21873	Abcam	WB 1:1000
Tubulin	#2144	Cell Signalling	WB 1:1000
Flag	F1804	Sigma	WB 1:1000
CHD4	Ab70469	Abcam	WB 1:1000
MTA1	A300-911A	Bethyl Laboratories	WB 1:1000
MTA2	Ab8106	Abcam	WB 1:1000, ChIP 5μg/IP
HDAC1	Ab7028	Abcam	WB 1:1000, ChIP 5μg/IP
HDAC2	39533	Active Motif	WB 1:1000
HDAC3	Ab7030	Abcam	WB 1:1000
MBD3	Ab157464	Abcam	WB 1:1000
FS2 (homemade)	N/A	N/A	ChIP 5μg/IP
H3K9Ac	07-352	Millipore	ChIP 5μg/IP
H3K27Ac	Ab4729	Abcam	ChIP 5μg/IP
NTD Pol2	14958	Cell Signalling	ChIP 15μl/IP
Pol II Ser2P	13499	Cell Signalling	ChIP 15μl/IP
Pol II Ser5P	13523	Cell Signalling	ChIP 15μl/IP

Supplementary Table 2. Primers

Primer usage	Forward	Reverse
PWWP2A full length (LIC cloning)	TACTTCCAATCCATGGCGGCCGTGGCTGCAGAG	TATCCACCTTTACTGTCACGTTTCAAACTGTGTCAACAAAGCCCG
PWWP2A 1-373 (LIC cloning)	TACTTCCAATCCATGGCGGCCGTGGCTGCAGAG	TATCCACCTTTACTGTCACCCATCCACTTTATGGTCAGTTTTCAG
PWWP2A Δ PWWP (LIC cloning)	TACTTCCAATCCATGGCGGCCGTGGCTGCAGAG	TATCCACCTTTACTGCTAGCCATCTGGTGTGACGCATTTAGAG
PWWP2A 148-373 (LIC cloning)	TACTTCCAATCCGGCGGGGACTCCACGGTGTCGC	TATCCACCTTTACTGTCACCCATCCACTTTATGGTCAGTTTTCAG
PWWP2A 148-649 (LIC cloning)	TACTTCCAATCCGGCGGGGACTCCACGGTGTCGC	TATCCACCTTTACTGCTAGCCATCTGGTGTGACGCATTTAGAG
PWWP2A 383-755 (LIC cloning)	TACTTCCAATCCCCTGCTGCCAAACGCGTTAAACTAGACAATGCTGTGGT TAAGGTTTCAAATATTGC	TATCCACCTTTACTGTCACGTTTCAAACTGTGTCAACAAAGCCCG
PWWP2A short isoform (LIC cloning)	TACTTCCAATCCATGGCGGCCGTGGCTGCAGAG	TATCCACCTTTACTGCTAATGTCTTTGTGCTGCTGAGCGGGTAGAG GTGCAG
PWWP2B full length (LIC cloning)	TACTTCCAATCCATGGAGCCGCGGGCC	TATCCACCTTTACTGTTACATTTCAAACTGG
CRISPR Guide1 (*pwwp2a*)	CACCGCGGCTTCGCTGCCGGGAAT	AAACATTCCCGGCAGCGAAGCCGC
CRISPR Guide2 (*pwwp2a*)	CACCGTTTGGAGCTGCATGGCGTC	AAACGACGCCATGCAGCTCCAAAC
CRISPR Guide3 (*pwwp2a*)	CACCGAACGGCCTTTTAGCCCGGC	AAACGCCGGGCTAAAAGGCCGTTC
CRISPR Guide4 (*pwwp2b*)	CACCGCGCCGACGGGTCCGCTTGA	AAACTCAAGCGGACCCGTCGGCGC
CRISPR Guide5 (*pwwp2b*)	CACCGTGATGTCAAGGACACGCGC	AAACGCGCGTGTCCTTGACATCAC
CRISPR PCR primers F1 + R1 (*pwwp2a*)	GGAGGAGGGAGAAACCAGCGAG	CTCCTCAGCCTCGGAGCTATCG
CRISPR PCR primers F2 + R2 (*pwwp2a*)	AGCCACTCTGGTATTGCCTAGAG	GGCGGCTGAGGTATTGATTGG
CRISPR PCR primers F3 + R3 (*pwwp2a*)	CACCAGATGGCAGGACCATATGTG	CACTTCAGGGGTCAGCTGC
CRISPR PCR primers F4 + R5 (*pwwp2b*)	CCTCTGGCTGACAACTCCTC	AGACACCTGGCCCACATAAG
Nanog promoter (ChIP-qPCR)	CAGCCGTGGTTAAAAGATGAATAAAG	GTAATGCAAAAGAAGCTGTAAGGTG
Nanog gene body (ChIP-qPCR)	GTAACACAATGAGACTGACTTGCTA	CGCATAGGATAGGAGGATAGGATAG
Acat2 promoter (ChIP-qPCR)	GATCATGTCGGGAAGGAGGG	CAGACAGGACAAGGCTGAG
Acat2 gene body (ChIP-qPCR)	AAAGAAGCCAAGTGTCTCTAAGAAG	AAGAGTGAAGAAGGCTGTAAGAATC
Neil2 promoter (ChIP-qPCR)	GGGTCCCGGGAAGATGTAC	GCCCTTGAGTCCAGGAAGTA
Neil2 gene body (ChIP-qPCR)	CAGGTTTGGTTTATTTGGCAGTATC	TCATAAACGTGATAGACCCCACTAG
Chr19 gene desert (ChIP-qPCR)	TGCATGAGCAGAGGACTAGG	AGAAGTGCAAGCTCAGAACCTT
