# Supplementary material for: A variant NuRD complex containing PWWP2A/B excludes MBD2/3 to regulate transcription at active genes
Source: Nat Commun. 2018 Sep 18;9:3798. doi: 10.1038/s41467-018-06235-9 (PMC6143588; doi:10.1038/s41467-018-06235-9)
Supplement: Supplementary file 3 — Description of Additional Supplementary Files [file 41467_2018_6235_MOESM3_ESM.pdf]

Description of Additional Supplementary Files

File Name: Supplementary Data 1
Description: H3K36me3 interactors.

File Name: Supplementary Data 2
Description: PWWP2A IP-MS in Hela.

File Name: Supplementary Data 3
Description: PWWP2A IP-MS in mESC.

File Name: Supplementary Data 4
Description: PWWP2B IP-MS in mESC.

File Name: Supplementary Data 5
Description: NGS summary.

File Name: Supplementary Data 6
Description: Mass spectrometry PRIDE summary.
